# The effect of metformin use on hypopharyngeal squamous cell carcinoma in diabetes mellitus patients

**DOI:** 10.1186/s12885-019-6083-5

**Published:** 2019-08-30

**Authors:** Yung-An Tsou, Wen-Dien Chang, Jian-Ji Lu, Tsu-Fang Wu, Hsiao-Ling Chen, Chuan-Mu Chen, Ming Hsui Tsai

**Affiliations:** 1 Department of Otolaryngology-Head and Neck Surgery, China Medical University Hospital, Taichung, Taiwan; 2 Department of Audiology and Speech-Language Pathology, Asia University, Taichung, Taiwan; 3 Department of Sport Performance, National Taiwan University of Sport, No.16, Sec. 1, Shuang-Shih Rd, Taichung, 40404 Taiwan; 4 Graduate Institute of Biomedicine Sciences, China Medical University, Taichung, Taiwan; 5 Biological Resources Department, Da-Yeh University, Changhua, Taiwan; 6 Department of Life Sciences, and Agricultural Biotechnology Center, National Chung Hsing University, Taichung, Taiwan

**Keywords:** Metformin, Hypopharyngeal cancer, Diabetes mellitus

## Abstract

**Background:**

Metformin is proven to improve the prognosis of various cancers, but it is unknown if metformin could ameliorate hypopharyngeal cancer in diabetes mellitus patients. This was a retrospective cohort study, and the effect and survival outcome of metformin on hypopharyngeal cancer with diabetes mellitus was investigated.

**Methods:**

There were 141 hypopharyngeal cancer patients collected in a tertiary referral center from December 1st, 2011 to December 31st, 2013. There were 49 patients without diabetes mellitus (DM) and 92 patients with DM. In the 92 DM patients, there were 43 patients with metformin used and 49 patients without metformin used. All received patients followed up until September 1st, 2015.

**Results:**

There was no significant difference in patients’ characteristics between the non-DM and DM groups, and also no significant difference in clinical T stage, N stage, metastatic condition, and disease stage between the non-DM and DM groups. DM with metformin patients had lower metastasis rates and better overall survival (OS) (*p* = 0.011) and disease-free survival (DFS) (*p* = 0.004) compared to non-DM and DM without metformin. Multivariate analysis also showed a better OS and DFS in DM-Met (+) with advanced hypopharyngeal cancer but not in early stage.

**Conclusion:**

There was less distant metastasis and better survival outcomes in hypopharyngeal cancer DM patients who use metformin.

## Background

Hypopharyngeal squamous cell carcinoma (HSCC) is usually diagnosed in the advanced stages with poor prognosis compared to other head and neck cancers [1, 2]. HSCC accounts for 3–5% of head and neck cancer patients [2]. The survival outcome is still poor after the improvement of surgical techniques or improvement of chemotherapy regiments and radiation technology, even if new trials for hypopharyngeal cancer treatment are ongoing such as cetuximab based radiotherapy (RT) [3] or induction chemotherapy followed by concurrent chemo-radiotherapy (CCRT) or surgery [4, 5].

Patients with diabetes mellitus (DM) have been reported to have higher incidence of oral cancer, oropharyngeal cancer, nasopharyngeal cancer, but not hypopharyngeal cancer [6, 7]. The better care control of DM leads to less complication and shorter admission duration [8, 9]. Some studies revealed cancer patients with DM have less cancer mortality after anti-glycemic regiment treatment [10, 11]. Literature reported that these patients with combinative metformin treatment has better overall survival and disease survival rate, suggesting potential anticancer roles for metformin [6]. Metformin use was reported to have better disease control in rectal and breast cancer [12, 13], and better survival outcomes in lung, colorectal cancer, and pancreatic cancer [10, 14, 15]. The increased response by metformin treatment was also reported in patients with esophagus cancer [16, 17].

Metformin rendered a better locoregional control in patients with advanced head and neck cancers (stage III–IV). Although metformin use was reported to have better survival outcomes in laryngeal cancer, there have been no reports of metformin treatment outcomes in hypopharyngeal cancers. Therefore, we conducted this cohort study to determine if metformin has anticancer functions in hypopharyngeal cancer in a tertiary referral center, China Medical University Hospital.

## Methods

### Study design and data collection

The approval of Institutional Review Boards of China Medical University Hospital (No. CMUH103-REC1–078), we reviewed the medical charts who received CCRT for hypopharyngeal cancer. From 2011 January to 2013 June, there were 141 patients enrolled in this cohort study. Demographic data, i.e. age, alcohol, betel nut, and smoking history, were recorded. In the medical charts, the clinical diagnosis results, rendered treatments, surgical interventions, and the associated dates were also reviewed and recorded. There were 49 patients with no DM, and 92 with DM. Among the 92 DM patients, there were 49 who used metformin OHA (oral hypoglycemic agents) for DM control, and 43 who used non-metformin OHA for DM control. The use of metformin was according to their previous OHA and persisted though the CCRT treatment until the latest follow up. Minimal follow up time was set 4 years. All patients with or without cisplatin-based chemotherapy underwent definitive RT, according to their disease status for organ preservation. The clinical TNM stage, age, gender, smoking, drinking, betel quid chewing, disease control, and survival outcomes, were all recorded as parameters.

### Statistical analysis

SPSS (version 21.0) was used to perform the statistical analyses by one researcher. Date from primary diagnosis to recurrence or death was recorded as disease-free survival (DFS), and date from primary diagnosis to last documented note or death was recorded as overall survival (OS). Kaplan-Meier analysis was used to estimate DFS and OS values, and log-rank test was used to compare the difference. Univariate analysis was performed using a Cox proportional hazards model. For between-group comparisons, continuous variable was performed using a chi-squared test, and category variable was performed using a t test. *P* values of all statistics were set at 0.05, and *p* < 0.05 as statistically significant.

## Results

There were 141 hypopharyngeal cancer patients with a mean age of 63.64 enrolled in this study, containing 49 non-DM patients (mean age = 63.28 ± 11.78) and 92 DM patients (mean age = 65.96 ± 11.27). All of them were treated by concurrent chemoradiation therapy (CCRT), treatment time is equal for all patients. The 30–35 fraction RT with total RT dosage 60–70 Gy (7–8 weeks duration), and chemotherapy regiment is cisplastin base drug on 3–6 courses (around 2–3 months duration) by the same treatment protocol. Of the patients, 57.45% had habits of drinking, 56.03% had habits of betel quid chewing, and 65.25% had habits of smoking. Briefly, 40 patients (28.37%) presented stage I-III stage cancer in early stage, and 88 patients (62.41%) presented stage IV stage cancer in advanced stage. There is no significant difference in age, alcohol drinking, betel quid chewing, or cigarette smoking between the non-DM and DM groups. There is also no significant difference in clinical T stage, N stage, metastatic condition, and disease stage between the non-DM and DM groups (Table 1).

**Table 1:** Patients characteristics (diabetic vs nondiabetic)

	All (*n* = 141)		Nondiabetes mellitus (*n* = 49)		Diabetes mellitus (*n* = 92)		
	No. of patients	(%)	No. of patients	(%)	No. of patients	(%)	p value
Age	63.64 y		63.28 y		65.96 y		
Alcohol	81	57.45	26	53.06	55	59.78	0.42
Betel nut	79	56.03	25	51.02	51	55.43	0.17
Cigarette	92	65.25	28	57.14	64	69.57	0.21
T1	15	10.64	7	14.29	8	8.70	0.11
T2	41	29.08	13	26.53	28	30.43	0.43
T3	35	24.82	11	22.45	24	26.09	0.14
T4	49	34.75	18	36.73	31	33.70	0.26
N0	30	21.28	8	16.33	22	23.91	0.23
N1	20	14.18	9	18.37	11	11.96	0.11
N2	87	61.70	30	61.22	57	61.96	0.13
N3	4	2.84	2	4.08	2	2.17	0.16
M0	131	92.91	47	95.92	84	91.30	0.11
M1	10	7.09	2	4.08	8	8.70	0.17
Early stage	40	28.37	13	26.53	27	29.35	0.39
Late stage	88	62.41	36	73.47	52	56.52	0.42

There were 92 hypopharyngeal cancer patients with DM, containing 43 DM patients without metformin treatment [DM-Met(−); mean age = 65.04 ± 9.76] and 49 DM patients with metformin treatment [DM-Met(+); mean age = 66.45 ± 12.34]. Comparing the groups of non-DM, DM-Met(−), and DM-Met(+), there is no significant difference in age, alcohol, betel quid habits, cigarette smoking, T stage, N stage, metastatic condition, or disease stage (Table 2).

**Table 2:** Patient characteristics (metformin users versus nonmetformin users)

	Nondiabetes mellitus (n = 49)		Diabetes mellitus met- (*n* = 43)		Diabetes mellitusmet+ (n = 49)		
	No. of patients	(%)	No. of patients	(%)	No. of patients	(%)	p value
Age	63.28 y		65.04 y		66.45 y		
Alcohol	26	53.06	27	62.79	28	57.14	0.46
Betel Nut	25	51.02	24	55.81	27	55.10	0.45
Cigarette	28	57.14	31	72.09	33	67.35	0.54
T1	7	14.29	2	4.65	6	12.24	0.57
T2	13	26.53	12	27.91	16	32.65	0.11
T3	11	22.45	14	32.56	10	20.41	0.15
T4	18	36.73	14	32.56	17	34.69	0.54
N0	8	16.33	9	20.93	13	26.53	0.51
N1	9	18.37	5	11.63	6	12.24	0.07
N2	30	61.22	27	62.79	30	61.22	0.17
N3	2	4.08	2	4.65	2	4.08	0.33
M0	47	95.92	35	81.40	49	100.00	0.33
M1	2	4.08	8	18.60	0	0.00	0.06
Early stage	13	26.53	11	25.58	16	32.65	0.17
Late stage	36	73.47	32	74.42	33	67.35	0.51

Met+, with metformin; met-, without metformin

**Fig. 1:**
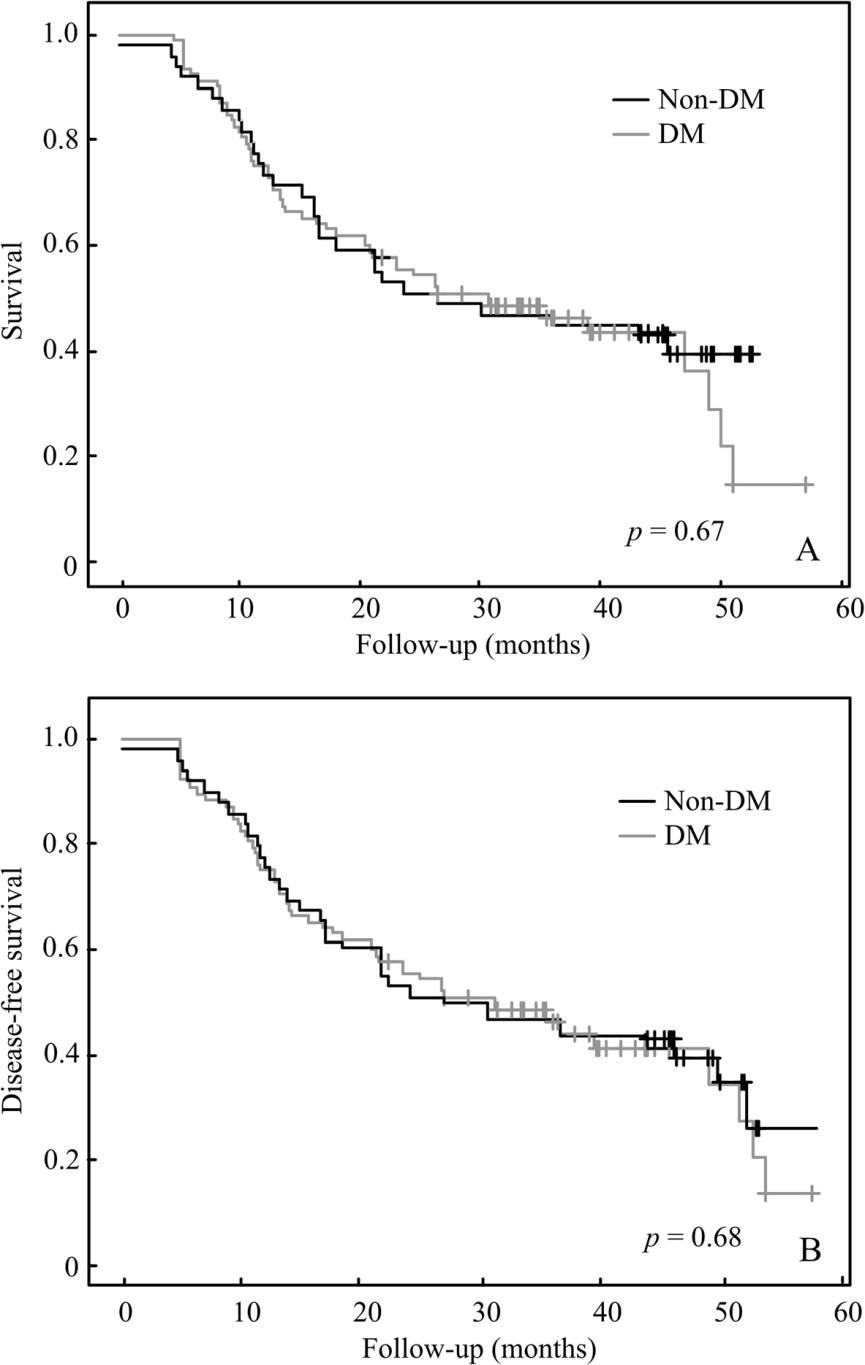
Impact on diabetes mellitus on overall survival (**a**) and disease-free survival (**b**)

**Fig. 2:**
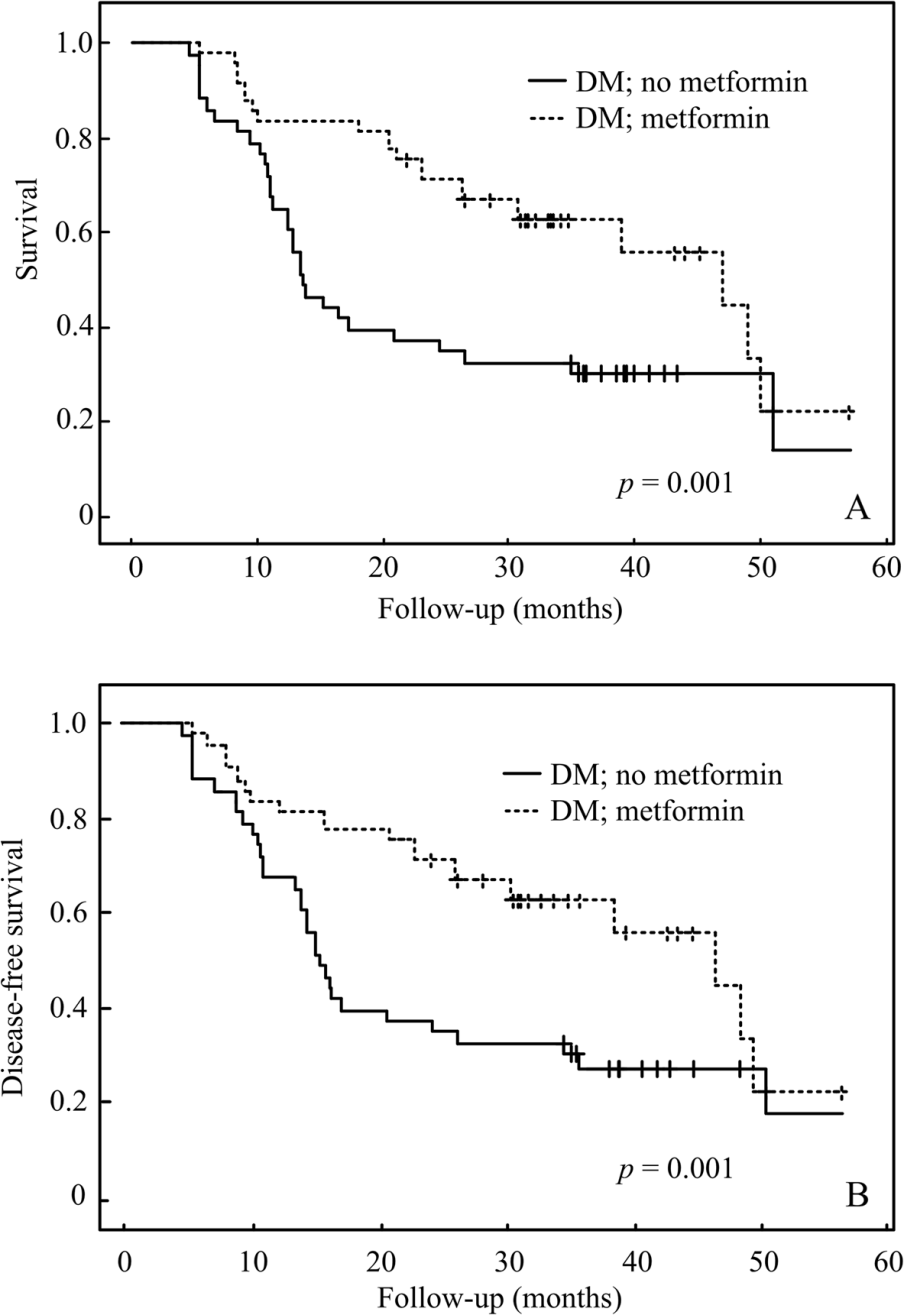
Kaplan-Meier analysis of overall survival (**a**) and disease-free survival (**b**) for metformin

The rates of OS and DFS for all patients at 4 years were 41.84 and 60.28%, respectively. There is no significant difference of OS and DFS between DM and non-DM patients (Fig. 1a and b, *p* = 0.67). There were best outcomes of OS and DFS in the DM-Met(+) group, followed by the no DM group, with the DM-Met(−) group producing the worst results (Fig. 2a, b). The OS at 4 years for the groups of DM-Met(+), and DM-Met(−) was 55.10, and 27.90%, respectively (*p* = 0.001) (Fig. 2a). The DFS at 4 years for the groups of DM-Met(+), and DM-Met(−) was 44.89, and 60.46%, respectively (p = 0.001) (Fig. 2b).

**Fig. 3:**
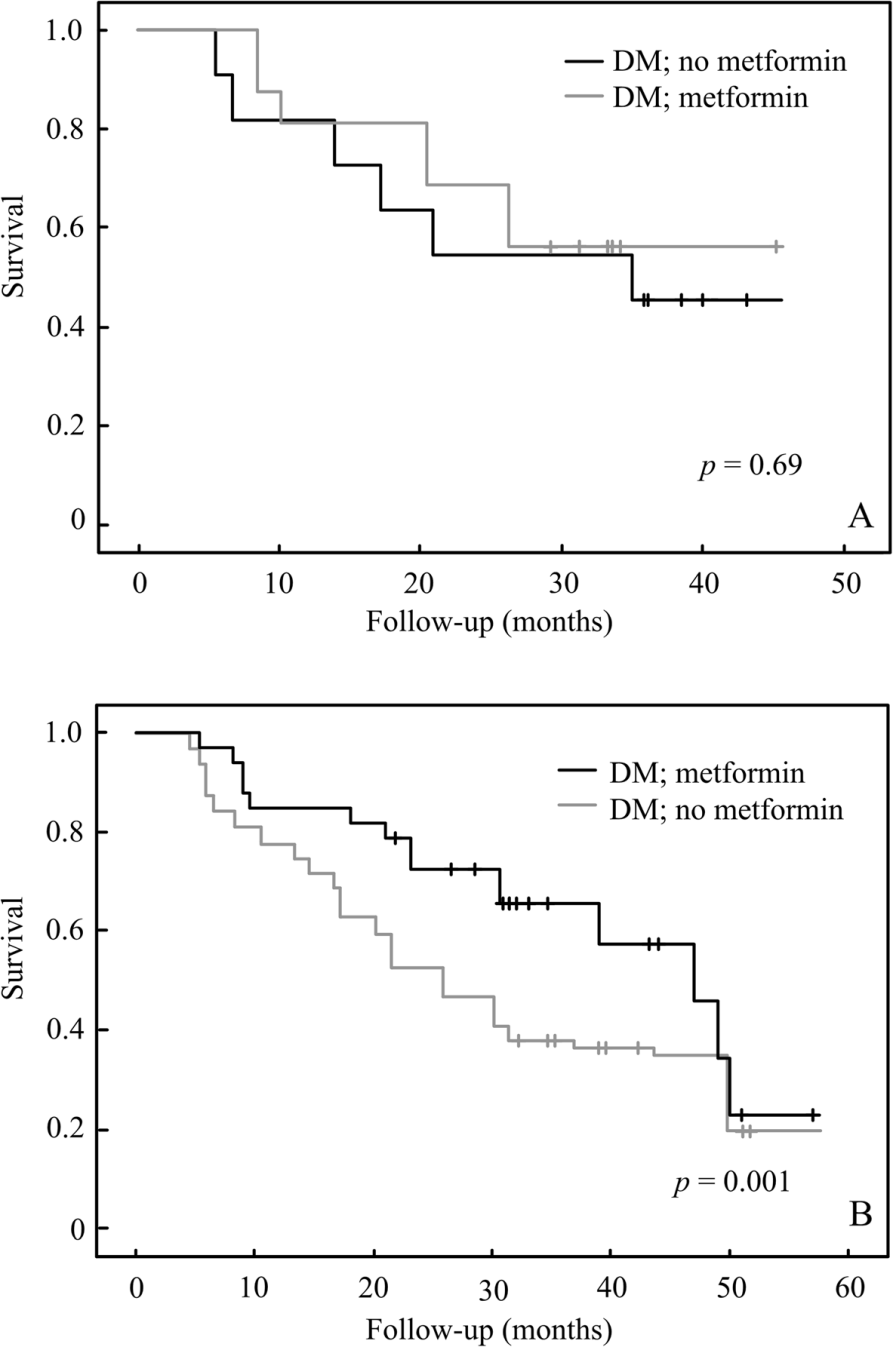
Kaplan-Meier analysis of overall survival on metformin in early (**a**) and late stage (**b**) of hypopharyngeal cancer patients

There was no significant difference in hemoglobin A1c values between the groups of DM-Met(+) and DM-Met(−), that is 6.81 vs 6.88, respectively. There was no significant difference in initiated TNM stage between the groups of DM-Met(+) and DM-Met. However, there was borderline lower metastasis in DM-Met(+) than DM-Met(−), which is 18.60% vs 0.00% (Table 2). There was no significant different in age (*p* = 0.57) between the groups of DM-Met(+) and DM-Met(−). Up to September 2015, 55.10, 32.43, and 40.48% of the patients in the groups of DM-Met(+), DM-Met(−), and non-DM were alive, respectively. Metformin is benefit to OS and DFS for hypopharyngeal cancer patients (Fig. 2). The metformin is also rendered a better disease specific survival in advanced hypopharyngeal DM patients in our cohort (Fig. 3). However it is not contributed to better survival outcome in early stage hypopharygeal DM patients.

Multivariate analysis showed that the group of DM-Met(+) has a better OS outcome than the group of DM-Met(−) in stage IV hypopharyngeal cancer (OR = 4.28, 95%CI = 1.45–12.65, *p* = 0.01). The DFS also showed a better outcome in the DM-Met(+) group than in the DM-Met(−) group (OR = 0.23, 95% CI = 0.07–0.68, p = 0.01) in Table 3.

**Table 3:** Multivariate analysis of overall survival and disease-free survival

Comparison	Overall survival		Disease-free survival	
	OR(95% CI)	p	OR(95% CI)	p
Early stage				
DM vs. non DM	1.67 (0.17–2.59)	0.56	1.25 (0.33–4.73)	0.73
DM (metformin) vs. DM (no metformin)	1.54 (0.32–7.22)	0.58	1.44 (0.09–2.14)	0.31
DM (no metformin) vs. non DM	0.62 (0.12–3.06)	0.55	1.75 (0.34–8.79)	0.49
DM (metformin) vs. non DM	1.95 (0.23–4.10)	0.96	1.78 (0.18–3.28)	0.73
Late stage				
DM vs. non DM	1.25 (0.53–2.93)	0.61	1.70 (0.29–1.67)	0.42
DM (metformin) vs. DM (no metformin)	4.28 (1.45–12.65)	0.01*	1.23 (0.07–0.68)	0.01*
DM (no metformin) vs. non DM	1.56 (0.18–1.66)	0.29	1.57 (0.52–4.71)	0.41
DM (metformin) vs. non DM	2.40 (0.91–6.35)	0.07	1.36 (0.13–0.98)	0.04*

* p < 0.05

## Discussion

In our study, we selected 49 of non-DM patients, and 92 of DM patients containing 43 of DM-Met(−) patients and 49 of DM-Met(+) patients. The percentile among non-DM, DM-Met(+), and DM-Met(−) was near equally distributed and all of these patients underwent RT base therapy for curative intent. In this retrospective cohort study of large non-surgical organ preservation, the DM-Met(+) group had better survival outcome than the other two groups. In head and neck cancer, hypopharyngeal cancer has the worst survival outcome2. It is hard to be diagnosed in the early stage, and the high locoregional or distant metastasis results in lower survival outcomes and poor disease control [2].

The combination of organ preservation therapy and chemoradiotherapy is widely accepted for patients with hypopharyngeal cancer. However, the poor prognosis is still happening in patients with hypopharyngeal cancer. This is because of how difficult to diagnose this cancer is in its early stage. Therefore, patients are often presented in the advanced stage. The other reason is these patients were found to have tumor resistance toward chemoradiotherapy. Thus, even with advances in treatment technologies such as intensity modulation radiation therapy (IMRT) and image-guided radiation therapy (IGRT), the survival rates are still poor. Moreover, the target therapy such as EGFR inhibitor, ie. Erbitux, is currently used as radiosensitizer for radiotherapy. However, the extreme costs lead to limited survival benefits [18]. Clinicians are still trying to find a radiosensitizer, and metformin is suggested to be the one of them.

Metformin was found to have benefits in treating various kinds of cancers, such as head and neck squamous cell carcinoma [6], colorectal cancer [12], breast cancer [13], pancreatic cancer [15], and prostate cancer [19]. It also improved distant metastasis-free survival in oropharyngeal cancer [20]. Several mechanisms were proven to explain its anticancer effects through direct or indirect insulin-dependent anticancer therapy. The animal study for oral squamous carcinoma also revealed tumor stasis and cell cycle arrest in G0/G1 phase, associating with activation of AMP kinase pathway to decrease cyclin D1, cyclin-dependent kinase 4/6 (CDK4/6) and phosphorylated retinoblastoma protein. Furthermore, metformin increased the apoptosis process by the down-regulation of Bcl-2 and Bcl-xL, as well as Bax upregulation [21]. A possible mechanism is that metformin blocks VEGF effect to decreased tumor neovasculization. Metformin has an antitumor angiogenesis effect by suppression of HER2/HIF-1α/VEGF pathway [22] and inhibits angiogenesis of hepatocellular carcinoma [23].

Some studies revealed its function to improve treatment response and use as a radio-sensitizer. Even though there has been reported that a better disease survival was possibly due to decreasing disease locoregional or distant metastasis in laryngeal and oropharyngeal cancer [20, 24]. Cell cycle arrest and apoptosis were found in salivary adenocarcinoma but not hypopharyngeal cancer with metformin treatment [25]. The real mechanisms of why metformin improves survival outcome and decreases metastatic condition are still unknown.

Metformin has been shown as a radiosensitizer in colorectal cancer by causing G2/M phase arrest [26], pancreatic cancer by inhibiting DNA repair to abrogate G2 phase checkpoint [27], esophagus cancer by activating ATM and AMPK [28], HCC by abrogating G2/M phase arrest [29]. However, there was no report on its role as a radiosensitizer in hypopharyngeal cancer by in vitro, in vivo, or clinical studies. Our studies also could not prove the radiosensitizing effect of metformin and need further human biochemical and flow cytometric analysis verified.

Even if the small sample size and non-random control trial could not precisely explain the mechanical effect of metformin, we still proved that metformin is beneficial to patients with hypopharyngeal cancer. The better survival outcomes were not observed in the early stage, but the outcomes were found in advanced disease status of hypopharyngeal cancer group. The possible explanation was smaller sample size in early stage patients or better disease control by RT. Also, we did not know concomitant oral hypoglycemic agents use or short supplementary courses of insulin use affect the efficacy, and this is the limitation of this study. The DM-Met(+) group had significantly better OS and DFS rates and a decreased disease metastasis rate in advanced hypopharyngeal cancer, however the larger prospective mechanical studies are still warranted in the future.

## Conclusions

Patients with advanced hypopharyngeal cell carcinoma taking metformin exhibited improved overall survival and better disease-free survival compared to non-metformin users, and even compared to patients that are not diabetic. The mechanisms of better sensitive to RT and less metastasis lead to improved clinical outcomes in human hypopharyngeal cancer are still warranted.

## Data Availability

All data generated or analysed during this study are included in this published article.

## Abbreviations

CCRT: Concurrent chemo-radiotherapy
DFS: Disease-free survival
DM: Diabetes mellitus
HSCC: Hypopharyngeal squamous cell carcinoma
IGRT: Image-guided radiation therapy
IMRT: Intensity modulation radiation therapy
OS: Overall survival; RT: Radiotherapy

## References

[1] Jang JY, Kim EH, Cho J, Jung JH, Oh D, Ahn YC, Son YI, Jeong HS (2016). Comparison of oncological and functional outcomes between initial surgical versus non-surgical treatments for hypopharyngeal cancer. Ann Surg Oncol.

[2] Cooper JS, Porter K, Mallin K, Hoffman HT, Weber RS, Ang KK, Gay EG, Langer CJ (2009). National Cancer Database report on cancer of the head and neck: 10-year update. Head Neck.

[3] Lefebvre JL, Pointreau Y, Rolland F, Alfonsi M, Baudoux A, Sire C, de Raucourt D, Malard O, Degardin M, Tuchais C, Blot E, Rives M, Reyt E, Tourani JM, Geoffrois L, Peyrade F, Guichard F, Chevalier D, Babin E, Lang P, Janot F, Calais G, Garaud P, Bardet E (2013). Induction chemotherapy followed by either chemoradiotherapy or bioradiotherapy for larynx preservation: the TREMPLIN randomized phase II study. J Clin Oncol.

[4] Edson MA, Garden AS, Takiar V, Glisson BS, Fuller CD, Gunn GB, Beadle BM, Morrison WH, Frank SJ, Shah SJ, Tao R, William WN, Weber RS, Rosenthal DI, Phan J (2016). Outcomes for hypopharyngeal carcinoma treated with organ-preservation therapy. Head Neck.

[5] Vourexakis Z, Janot F, Dulguerov P, Le Ridant AM (2014). Larynx preservation protocols: long-term functional outcomes in good responders to induction chemotherapy for pyriform sinus carcinoma. ORL J Otorhinolaryngol Relat Spec.

[6] Rego DF, Pavan LM, Elias ST, De Luca Canto G, Guerra EN (2015). Effects of metformin on head and neck cancer: a systematic review. Oral Oncol.

[7] Tseng KS, Lin C, Lin YS, Weng SF (2014). Risk of head and neck cancer in patients with diabetes mellitus: a retrospective cohort study in Taiwan. JAMA Otolaryngol Head Neck Surg.

[8] Raikundalia MD, Fang CH, Spinazzi EF, Vazquez A, Park RC, Baredes S, Eloy JA (2016). Impact of diabetes mellitus on head and neck cancer patients undergoing surgery. Otolaryngol Head Neck Surg.

[9] Tsou YA, Hua CH, Lin MH, Tseng HC, Tsai MH, Shaha A (2010). Comparison of pharyngocutaneous fistula between patients followed by primary laryngopharyngectomy and salvage laryngopharyngectomy for advanced hypopharyngeal cancer. Head Neck.

[10] Menamin UC, Cardwell CR, Hughes CM, Murray LM (2016). Metformin use and survival from lung cancer: a population-based cohort study. Lung Cancer.

[11] Franciosi M, Lucisano G, Lapice E, Strippoli GF, Pellegrini F, Nicolucci A (2013). Metformin therapy and risk of cancer in patients with type 2 diabetes: systematic review. PLoS One.

[12] He XK, Su TT, Si JM, Sun LM (2016). Metformin is associated with slightly reduced risk of colorectal cancer and moderate survival benefits in diabetes mellitus: a meta-analysis. Medicine.

[13] El-Haggar SM, El-Shitany NA, Mostafa MF, El-Bassiouny NA (2016). Metformin may protect nondiabetic breast cancer women from metastasis. Clin Exp Metastasis.

[14] Saber MM, Galal MA, Ain-Shoka AA, Shouman SA (2016). Combination of metformin and 5-aminosalicylic acid cooperates to decrease proliferation and induce apoptosis in colorectal cancer cell lines. BMC Cancer.

[15] Ambe CM, Mahipal A, Fulp J, Chen L, Malafa MP (2016). Effect of metformin use on survival in resectable pancreatic cancer: a single-institution experience and review of the literature. PLoS One.

[16] Skinner HD, McCurdy MR, Echeverria AE, Lin SH, Welsh JW, O'Reilly MS, Hofstetter WL, Ajani JA, Komaki R, Cox JD, Sandulache VC, Myers JN, Guerrero TM (2013). Metformin use and improved response to therapy in esophageal adenocarcinoma. Acta Oncol.

[17] Sekiguchi RT, Pannuti CM, Silva HT, Medina-Pestana JO, Romito GA (2012). Decrease in oral health may be associated with length of time since beginning dialysis. Spec Care Dentist.

[18] Magrini SM, Buglione M, Corvò R, Pirtoli L, Paiar F, Ponticelli P, Petrucci A, Bacigalupo A, Crociani M, Lastrucci L, Vecchio S, Bonomo P, Pasinetti N, Triggiani L, Cavagnini R, Costa L, Tonoli S, Maddalo M, Grisanti S (2016). Cetuximab and radiotherapy versus cisplatin and radiotherapy for locally advanced head and neck cancer: a randomized phase II trial. J Clin Oncol.

[19] Zhang T, Zhang L, Zhang T, Fan J, Wu K, Guan Z, Wang X, Li L, Hsieh JT, He D, Guo P (2014). Metformin sensitizes prostate cancer cells to radiation through EGFR/p-DNA-PKCS in vitro and in vivo. Radiat Res.

[20] Spratt DE, Beadle BM, Zumsteg ZS, Rivera A, Skinner HD, Osborne JR, Garden AS, Lee NY (2016). The influence of diabetes mellitus and metformin on distant metastases in oropharyngeal cancer: a multicenter study. Int J Radiat Oncol Biol Phys.

[21] Luo Q, Hu D, Hu S, Yan M, Sun Z, Chen F (2012). In vitro and in vivo anti-tumor effect of metformin as a novel therapeutic agent in human oral squamous cell carcinoma. BMC Cancer.

[22] Wang J, Li G, Wang Y, Tang S, Sun X, Feng X, Li Y, Bao G, Li P, Mao X, Wang M, Liu P (2015). Suppression of tumor angiogenesis by metformin treatment via a mechanism linked to targeting of HER2/HIF-1alpha/VEGF secretion axis. Oncotarget.

[23] Qu H, Yang X (2015). Metformin inhibits angiogenesis induced by interaction of hepatocellular carcinoma with hepatic stellate cells. Cell Biochem Biophys.

[24] Sandulache VC, Hamblin JS, Skinner HD, Kubik MW, Myers JN, Zevallos JP (2014). Association between metformin use and improved survival in patients with laryngeal squamous cell carcinoma. Head Neck.

[25] Guo Y, Yu T, Yang J, Zhang T, Zhou Y, He F, Kurago Z, Myssiorek D, Wu Y, Lee P, Li X (2015). Metformin inhibits salivary adenocarcinoma growth through cell cycle arrest and apoptosis. Am J Cancer Res.

[26] Jeong YK, Kim MS, Lee JY, Kim EH, Ha H (2015). Metformin radiosensitizes p53-deficient colorectal cancer cells through induction of G2/M arrest and inhibition of DNA repair proteins. PLoS One.

[27] Wang Z, Lai ST, Ma NY, Deng Y, Liu Y, Wei DP, Zhao JD, Jiang GL (2015). Radiosensitization of metformin in pancreatic cancer cells via abrogating the G2 checkpoint and inhibiting DNA damage repair. Cancer Lett.

[28] Feng T, Li L, Ling S, Fan N, Fang M, Zhang H, Fang X, Lan W, Hou Z, Meng Q, Jin D, Xu F, Li Y (2015). Metformin enhances radiation response of ECa109 cells through activation of ATM and AMPK. Biomed Pharmacother.

[29] Kim EH, Kim MS, Cho CK, Jung WG, Jeong YK, Jeong JH (2014). Low and high linear energy transfer radiation sensitization of HCC cells by metformin. J Radiat Res.

